# Recurrent *FAN1* p.W707X Pathogenic Variant Originating Before ad 1800 Underlies High Frequency of Karyomegalic Interstitial Nephritis in South Pacific Islands

**DOI:** 10.1016/j.ekir.2021.05.010

**Published:** 2021-05-21

**Authors:** Lorraine Gueguen, Ronan Delaval, Maud Blanluet, Hervé Sartelet, Sylvie Leou, Catherine Dubois d’Enghien, Lisa Golmard, Dominique Stoppa-Lyonnet, Pascale Testevuide, Stanislas Faguer

**Affiliations:** 1 Service de Néphrologie, Centre Hospitalier du Taaone, Papeete, French Polynesia; 2 Service de Génétique, Institut Curie, Paris, France; 3 Service d’anatomopathologie, Centre Hospitalier Universitaire de Nancy, Vandoeuvre-les-Nancy, France; 4 Université de Paris, Sciences et Lettres, Paris, France; 5 INSERM U830, Institut Curie, Paris, France; 6 Université de Paris, Paris, France; 7 Département de Néphrologie et Transplantation d’Organes, Centre National de Référence des Maladies Rénales Rares, Centre Hospitalier Universitaire de Toulouse, Toulouse, France; 8 Institut National de la Santé et de la Recherche Médicale, UMR 1297, Institut des Maladies Métaboliques et Cardiovasculaires, Toulouse, France; 9 Université Paul Sabatier–Toulouse-3, Toulouse, France

**Keywords:** bronchiectasis, *FAN1*, karyomegaly, nephropathy, South Pacific Islands

## Introduction

Karyomegalic interstitial nephritis (KMIN; OMIM 814617, ORPHA 401996) is a very rare autosomal recessive inherited systemic disease caused by germline pathogenic variants in the *FAN1* gene, which encodes the FANCD2/FANCI-associated nuclease 1.^1^ Kidneys are involved in most if not all patients with KMIN, with characteristics of chronic tubulointerstitial nephropathy at presentation. Kidney pathology mainly shows interstitial infiltration of inflammatory cells and fibrosis, tubular atrophy, tubular membrane degeneration, and unusual enlarged nuclei of tubular cells.^1^, ^2^, ^3^

KMIN was first described in 1974 in a young woman with liver carcinoma and interstitial nephritis with dysplastic tubules.^4^ In rare patients, lung disease is at the forefront and may culminate in severe respiratory failure requiring lung transplantation,^5^ but extrarenal symptoms are often absent or mild (recurrent upper respiratory tract infections and abnormal liver tests) even if tissue examination of lung, heart, brain, gut, vessels, skin, or thyroid frequently show pathologic changes.^3^ In contrast, progression toward chronic kidney disease (CKD) and end-stage renal disease before 50 years of age is the rule.^3^

Germline pathogenic variants in the *FAN1* gene were recently identified in patients with KIN with *FAN1* linking CKD to defective DNA repair in tubular cells.^1^ To date, fewer than 60 patients with KMIN have been reported worldwide, including 14 patients with proven *FAN1* pathogenic variant.^1^^,^^3^^,^^6^^,^^7^ Both truncating and missense pathogenic variants were described, and no common pathogenic variant was identified. Incidence of KMIN is estimated at <1/1,000,000 inhabitants.

Here, we report the high prevalence of KMIN in French Polynesia, a large territory covering 188 islands spread over 4167 km^2^ in the eastern South Pacific and including ≈280,000 inhabitants (Polynesians ≈90%). The 12 affected patients from 5 families were homozygous for the *FAN1* c.2120G>A, p.(W707X) pathogenic variant, previously identified in 2 siblings without reported consanguinity and of Maori descent from New Zealand.^1^

## Results

### Patients

KMIN was identified in 12 Polynesian patients born in islands from various archipelagos of the French Polynesia (Supplementary Figure S1). Diagnosis relied on genetic testing in 8 individuals. In the remaining 4, diagnosis relied on a suggestive phenotype and a first-degree relative with biopsy-proven karyomegaly and a positive genetic testing of *FAN1*. In the 8 tested patients, genetic analysis identified a recurrent homozygous *FAN1* c.2120G>A, p.(W707X) variant that was classified as pathogenic according to the American College of Medical Genetics and Genomics (ACMG) guidelines as it leads to a premature termination codon in the exon 8 of *FAN1*, which contains 15 exons, and it is absent in individuals from the Genome Aggregation Database (gnomAD), v2.1.1, whereas it has been identified in several patients with KMIN.S1 This variant had been previously observed with a homozygous status in 2 brothers with KMIN living in New Zealand.^1^ Cosegregation analysis also supported the variant pathogenicity as all affected patients were homozygous for this variant, whereas among the 13 asymptomatic tested relatives, 10 individuals were heterozygous and 3 individuals did not carry the variant.

### Kidney Presentation and Outcomes

**Figure 1:**
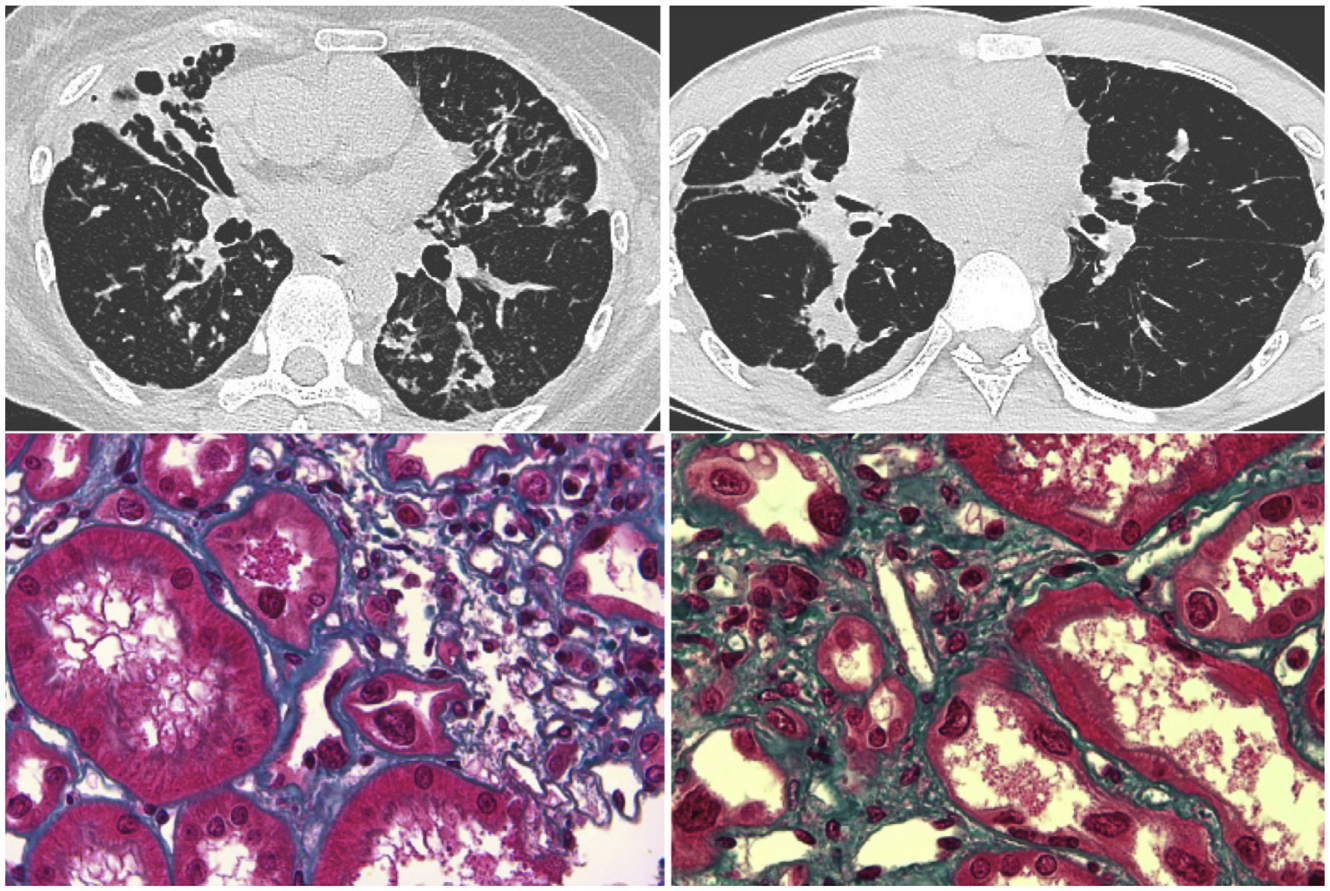
Pathologic changes and imaging. Upper left and right. Chest computed tomographic scan: bronchiectasis. Lower left and right: enlarged nuclei of tubular cells and interstitial fibrosis (Masson Trichrome staining, original magnification ×24).

Median age at recognition of the kidney disease was 32 years (interquartile range 18–56) (Table 1). At diagnosis, estimated glomerular filtration rate (GFR) was 33 mL/min per 1.73 m^2^ (interquartile range 3–62). Kidney presentation consisted of CKD with absent or mild tubular proteinuria (<1 g/d) and bland urinalysis. Four patients had hypertension, and none had electrolyte disorder. Renal ultrasonography (US) showed bilateral renal atrophy in 7 patients (58%), whereas kidney size was normal in the others. None had renal cyst or malformation. Kidney biopsy (*n*=7) showed typical features of tubulointerstitial nephropathy. Glomeruli were normal except for glomerulosclerosis in the fibrosis area. Interstitial fibrosis with atrophic tubules and tubular dilatation was common. Karyomegalic cells were observed in all samples with massively enlarged nuclei (3–4-fold the normal size) (Figure 1). No viral inclusion was identified, and SV40 immunostaining was negative ruling out a BK virus–associated nephropathy.

**Table 1:** Clinical characteristics and outcomes

Pt.	Sex	Age, yr	*FAN1* genotype	Kidney presentation					Last follow-up		Liver	ENT infections	Lung disease	Others
				eGFR	Pu	Renal biopsy	High BP	Kidney size	Age	eGFR				
F1.IXg	F	56	c.2021G>A (Homoz.)	33	0.22	—	Yes	9	73	ESRD	Chol. CS	Yes	No	No
F1.Xa	F	31	c.2021G>A (Homoz.)	25	0.23	KMIN	Yes	8	34	12	Chol. CS	No	LF	No
F1.Xb	M	18	c.2021G>A (Homoz.)	62	0.35	KMIN	No	8.5	27	ESRD	Chol. CS	No	No	Heart
F1.Xj	M	31	c.2021G>A (Homoz.)	33	0.22	KMIN	No	8.5	33	ESRD	Chol.	No	Bronch., ILD	Heart
F2.IIIh	M	29	—	44	0.30	KMIN	No	10	32	ESRD	Chol.	Yes	Bronch.	No
F2.IIIi	M	37	c.2021G>A (Homoz.)	3	0.50	—	No	8	40	ESRD	Chol.	Yes	Bronch., ILD	No
F3.IIa	M	39	c.2021G>A (Homoz.)	72	0.40	KMIN	Yes	10	49	16	—	No	Bronch.	No
F4.IIa	M	32	c.2021G>A (Homoz.)	28	0.75	KMIN	No	10	32	27	Chol. Cyt.	No	Bronch., LTI	No
F5.IIb	F	32	—	ESKD	—	—	No	—	32	ESRD	Chol.	—	Bronch.	No
F5.IIc	F	31	—	15	0.4	—	Yes	7	43	ESRD	Chol.	—	Bronch., ILD	No
F5.IIe	F	35	—	54	0.5	—	No	8	45	ESRD	Chol.	No	Bronch., ILD	No
F5.IIg	M	41	c.2021G>A (Homoz.)	18	0.8	KMIN	No	10	42	12	Cyt.	No	Bronch.	No

BP, blood pressure; Bronch., bronchiectasis; Chol, cholestasis; CS, choledochus stenosis; Cyt, cytolysis; eGFR, estimated glomerular filtration rate; ENT, ear, nose, throat; ESRD, end-stage renal disease; FU, follow-up; ILD, interstitial lung disease; KMIN, karyomegalic interstitial nephritis; LF, lung fibrosis; LTI, lower tract infection; Pt, patient name; Pu, proteinuria.

Eight patients (75%) developed end-stage kidney disease, including 5 before 40 years of age. The oldest patient reached end-stage kidney disease at the age of 71 years. From the diagnosis of kidney disease, the median yearly decline of eGFR was –7 mL/min per 1.73 m^2^ (–1.5 to –11 mL/min per 1.73 m^2^). Two patients received kidney transplantation without relapse of the kidney karyomegaly.

### Extrarenal Phenotype

Three patients had recurrent upper airways infection (sinusitis, otitis, laryngitis), 5 developed at least 1 episode of pneumonia or bronchiolitis and 3 developed lung tuberculosis. KMIN had a primary pulmonary presentation in 3 patients. The main computed tomographic scan abnormalities were pleural thickening, bronchiectasis, and lung fibrosis (Figure 1).

All the patients had nonicteric cholestasis (γ-glutamyltransferase 3–15 times the upper limits of normal) and 2 had elevation of aspartate- and alanine-aminotransferases. No patient developed portal hypertension or liver failure. Liver ultrasonography was normal in all, but magnetic resonance imaging showed nonobstructive segmental stenosis of the choledochus or intrahepatic biliary ducts in 3 and 2 patients, respectively. Liver biopsy (*n*=3) did not show overt karyomegalic cells or liver fibrosis. The pancreas was normal.

One patient receiving chronic renal replacement therapy developed severe hypertrophic cardiomyopathy with features of karyomegaly on heart biopsy. Two patients had hypothyroidism, including one with antithyroglobulin antibodies. Retinal examination (*n*=3) was normal, as well as bone marrow examination (*n*=1). No patient developed solid or hematologic cancer.

### Phenotypes in Heterozygous Individuals

No individual carrying the *FAN1* c.2120G>A, p.(W707X), pathogenic variant with a heterozygous status developed CKD or abnormal urinalysis, suggesting that they theoretically can be considered as potential living kidney donors for their relatives. One individual developed a nonicteric cholestasis related to nonalcoholic steatosis. One male patient developed cholangiocarcinoma. None developed colon carcinoma or other cancers.

### Familial Linkage and Variant Prevalence

After genealogic investigations, all individuals could be placed in pedigrees of 5 unrelated families. In family 1, although most cases belonging to this large family was formerly considered as unrelated, parental links could be identified and pedigree was defined up to 11 generations. The oldest ancestor was born in ad 1648 and genealogical tree suggested that the founder effect occurred before ad 1800 (i.e., before the F1-VII generation; Supplementary Figure 1). Despite extensive familial researches, no link between the various branches of family 1 could be identified, thus suggesting a date of the founder effect even older.

The structure of the family 1 pedigree with unrelated individuals necessarily heterozygous for the *FAN1* p.(W707X) pathogenic variant (e.g., individual F1-IX-k) but also the identification of KMIN in families in whom the disease was identified in only 1 generation, both suggesting that this variant spread in the Polynesian population. Consistently, among 7 patients awaiting for kidney transplantation because of end-stage kidney disease of unknown origin (no kidney biopsy) and not related to the 5 families described here, genetic testing for the *FAN1* p.(W707X) pathogenic variant was positive in 1 individual with a heterozygous status. Thus, the *FAN1* p.(W707X) pathogenic variant is frequently identified in Polynesian individuals and also present in New Zealand,^1^ another island of South Pacific, but has never reported in other parts of the world.

## Discussion

In this study, we identified KMIN as a frequent disease in French Polynesia, a territory located in southwest Pacific and characterized by insularity. Twelve patients from 5 families (≈1 case for 24,000 individuals) were identified compared with fewer than 60 cases described to date in the world (incidence below 1 case per 1,000,000 individuals). Because CKD and its usual risk factors (diabetes mellitus, hypertension, and obesity) are highly prevalent in French Polynesia, and kidney biopsy is not systematic, incidence of KMIN was likely underestimated in our study.

By analyzing the genealogical tree of a large family with KMIN, we could show that the founder event that subsequently led to the spreading of the *FAN1* p.(W707X) pathogenic variant in French Polynesia probably occurred before ad 1800. Interestingly, this variant was also identified in 2 brothers of Maori descent born in New Zealand,^1^ whereas all *FAN1* pathogenic variants described to date are unique to each family.^1^^,^^3^ Recent knowledges defined how New Zealand was colonized over centuries from Polynesian ancestors.S2 We hypothesize that a founder effect may have led to an increased prevalence of this pathogenic variant in Polynesia and subsequently in other South Pacific Islands but comparison between *FAN1* haplotypes of these Maori patients and those included in our series could not be performed. Last, our study clearly demonstrates how rare inherited diseases or genetic variants can be over-represented in area characterized by a small-sized population and insularity.

The identification of a frequent *FAN1* founder pathogenic variant in French Polynesians also furnished a unique opportunity to address the respective roles of genotype and environment in the severity scaling of KMIN. As previously described, most patients with KMIN will reach end-stage kidney disease, but age at dialysis onset varied from 27 to >70 years, even in the same family and despite a common *FAN1* genotype.^3^ This was reminiscent of *in vitro* findings showing that *FAN1*-deficient cells have hypersensitivity to DNA damages and that environmental genotoxins promote kidney fibrosis in patients with KMIN.^8^^,^^9^ DNA damages are mostly secondary to acute or chronic exposure to environmental toxins, radiations, or to ageing, and various exposure may explain a part of the CKD progression heterogeneity. In our cohort, all patients came from French Polynesia but lived in various islands and were thus exposed to different risks of subclinical kidney insults. We could not identify a specific nephrotoxin, but these findings reinforce the role of environment in the development of CKD and highlights the complex interplay between kidney epithelium homeostasis, injuries, and repair mechanisms. Whether interindividual variations to radioactivity exposure (here, related to potential contaminations of some areas of French Polynesia following atmospheric nuclear tests in the second part of the 20th century) may explain kidney outcomes heterogeneity warrants further explorations. Because kidney biopsy was only performed at the time the patient was referred for, whether the progression of karyomegalic cell numbers within kidneys correlates with kidney outcomes could not be addressed. Considering the risk of disease transmission in case of kidney transplantation with living donation from a brother or sister with overt KMIN, our findings suggest ruling out homozygous *FAN1* variations in donors but not to exclude those with heterozygous status.

Extra-kidney phenotype was more homogeneous, especially for liver involvement that was almost constant and lung phenotype that was symptomatic in 10 of 12 patients. Lung and ear, nose, and throat phenotypes were poorly specific and characterized by frequent infection during infancy. Bronchiectasis and interstitial lung diseases were common, but sometimes only detectable using chest computed tomographic scan, highlighting the need of careful phenotyping facing kidney disease with tubulointerstitial presentation. Whereas severe kidney failure was the rule, no patient developed chronic lung or liver failure. Chronic cholestasis was frequently observed and associated with severe pruritus in one of our patients, but karyomegalic cells were rarely seen within the liver. KMIN should be searched for using kidney biopsy in patients with CKD of unknown origin and concomitant cholestasis or bronchiectasis.

Finally, among the individuals with a homozygous or heterozygous status for the FAN1 variation, none developed solid cancer, as previously described.S3

In summary, we report here the high incidence of karyomegalic interstitial nephritis in French Polynesia owing to a recurrent *FAN1* pathogenic variant with a founder effect that occurred more than 200 years ago. The heterogeneity of the glomerular filtration rate slope despite a common genotype supports the role of modifying genetic factors or environment and genotoxins in *FAN1*-related nephropathy.

## Disclosure

All the authors declared no competing interests.

## Author Contributions

SF, PT, LG, and RD designed the study; LG and SF collected and analyzed the data; MB, CD, LG, and DSL performed genetic analyses; HS performed kidney pathology; SF, DSL, and LG wrote the manuscript; all the authors approved the final version of the manuscript.

## Supplementary Material

Supplementary File (PDF)

**Figure S1.** Genealogical trees of family 2 to 5.

**Strobe list**.
