# Supplementary material for: Recurrent FAN1 p.W707X Pathogenic Variant Originating Before ad 1800 Underlies High Frequency of Karyomegalic Interstitial Nephritis in South Pacific Islands
Source: Kidney Int Rep. 2021 May 21;6(8):2207–11. doi: 10.1016/j.ekir.2021.05.010 (PMC8344115; doi:10.1016/j.ekir.2021.05.010)
Supplement: Supplementary File (PDF) [file mmc1.pdf]

Patients and methods

In this retrospective observational study, we included all the patients followed in the Nephrology Department of the Taoone Hospital (Tahiti, French Polynesia, France) and who had molecular screening of *FAN1*. First-degree relatives of patients with proven *FAN1* pathogenic variant were also included, even in the absence of molecular screening. Diagnosis of KIN relied on (*i*) the identification of karyomegalic cells on renal biopsy with a positive genetic testing of *FAN1*, or (*ii*) a positive genetic testing or a phenotype compatible with KIN in first-degree relatives of a patient fulfilling the first criteria. A suggestive phenotype was defined by the association of chronic kidney disease with tubule-interstitial presentation (with or without renal biopsy), chronic liver tests abnormalities and diffuse interstitial lung disease or bronchiectasis of unknown origin. The study was conducted according to Declaration of Helsinki, as revised in 2004, and the French law regarding retrospective observational studies. All patients gave written informed consent.

The following laboratory values were collected at presentation and during follow-up: serum creatinine, estimated glomerular filtration rate using the CKD-EPI formula, electrolytes, urinary protein to creatinine ratio (uPCr), urinalysis, liver tests. Chronic kidney disease was staged according to the KDIGO classification. Lung (CT-scan), liver (MRI) and renal imaging (ultrasonography, CT-scan) were also reviewed.

Continuous variables were given as the median and ranges, while categorical variables were given as number and percentage

Genetic analysis of the *FAN1* whole coding sequence and exon-intron junctions was performed for index cases by next generation sequencing, with SureSelect QXT enrichment (Agilent) and sequencing on NextSeq 500 (Illumina) according to manufacturers' instructions. *FAN1* targeted genetic testing for relatives was performed by Sanger sequencing.

Supplementary Figure 1: Genealogical trees. Patients with the p.W707X pathogenic variation at the homozygous status are shown in dark (+/+), at the heterozygous status in grey (+/-) and not tested or with normal FAN1 in white (-/-).

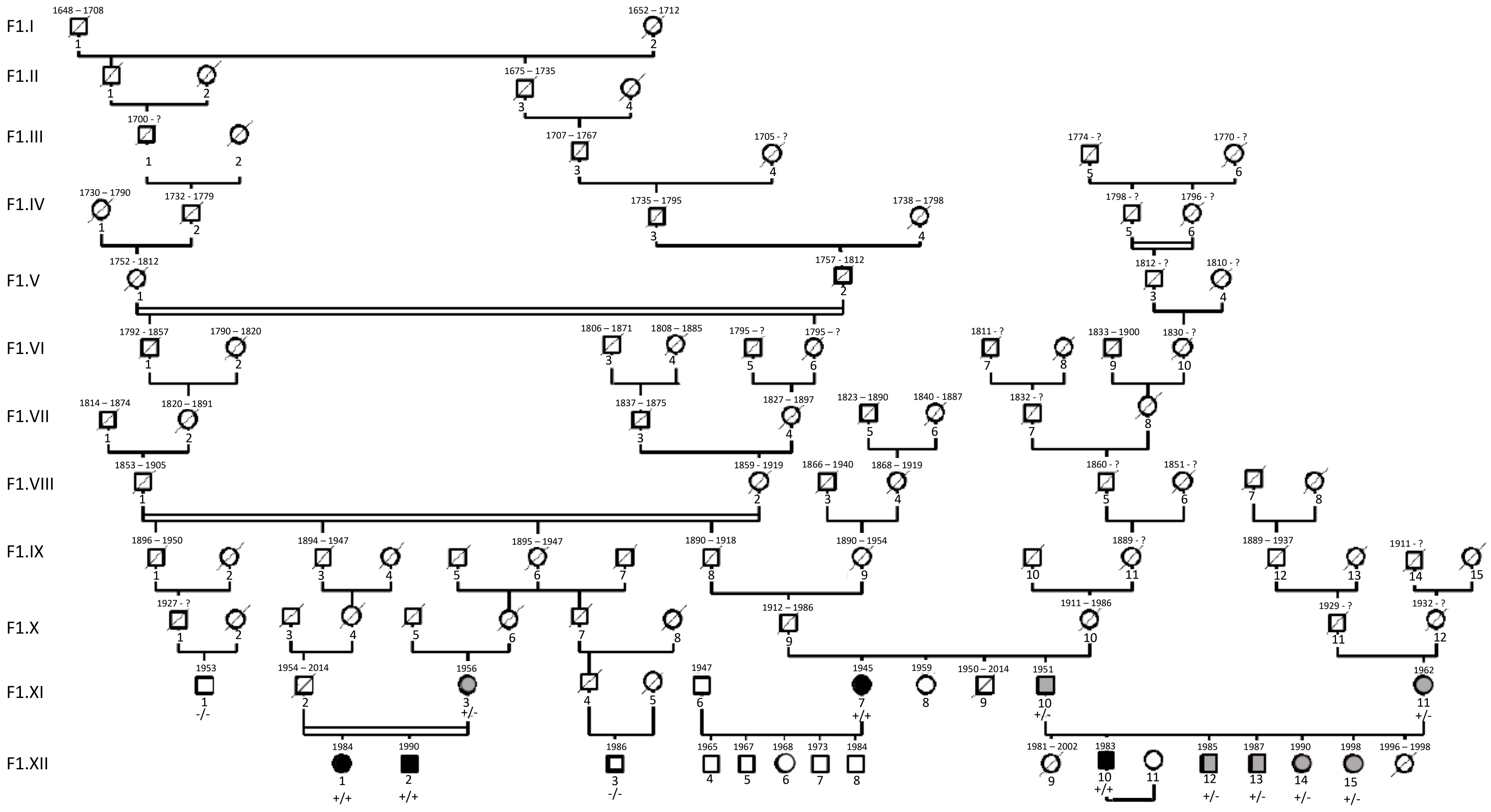
F1.I
F1.II
F1.III
F1.IV
F1.V
F1.VI
F1.VII
F1.VIII
F1.IX
F1.X
F1.XI
F1.XII

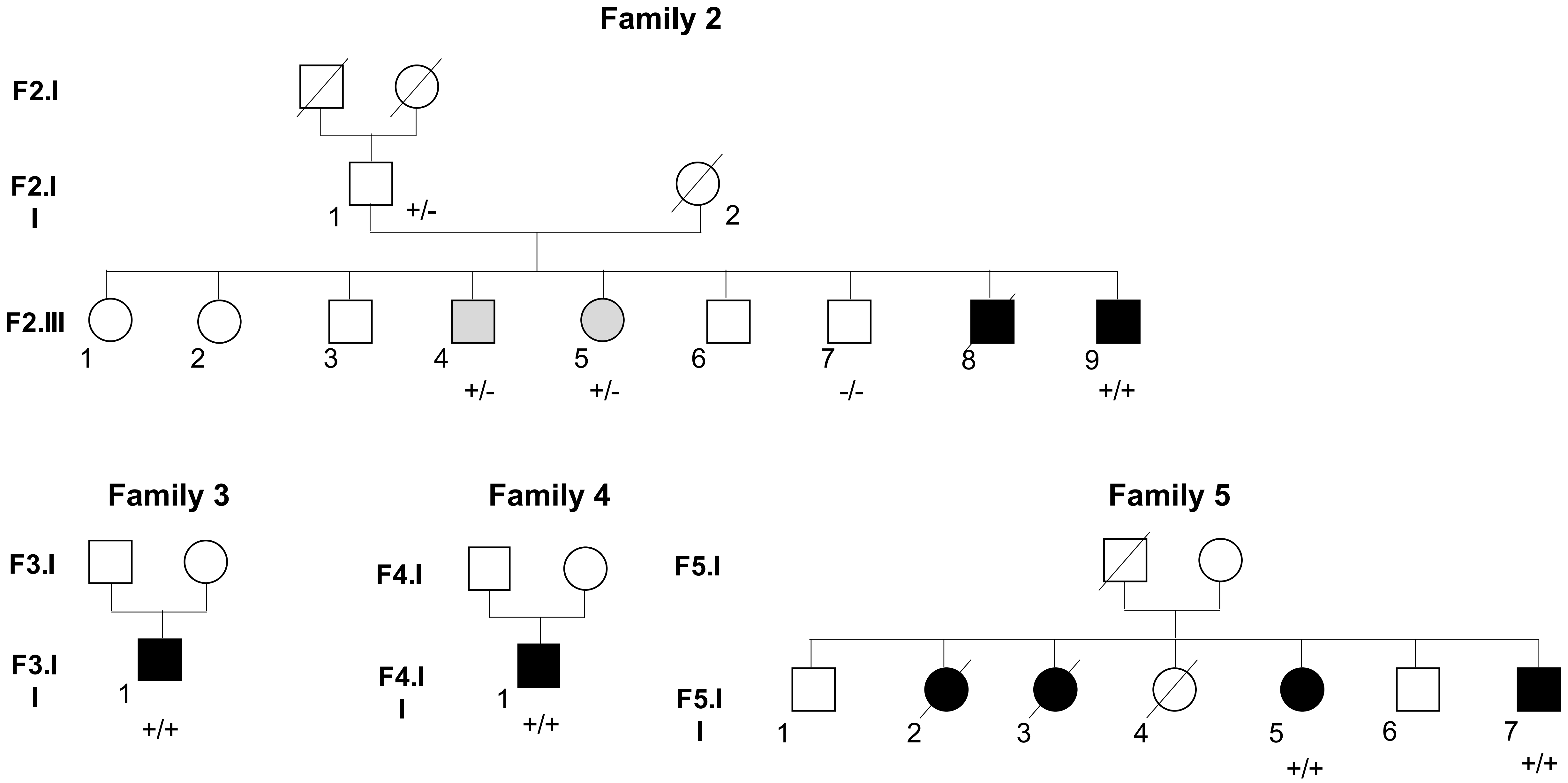

Family 2
F2.I
F2.II
1 +/-
2
F2.III
1
2
3
4 +/-
5 +/-
6
7 -/-
8
9 +/+
Family 3
F3.I
F3.II
1 +/+
Family 4
F4.I
F4.II
1 +/+
Family 5
F5.I
F5.II
1
2
3
4
5 +/+
6
7 +/+

Ref. S1: Richards S, Aziz N, Bale S et al. Standards and guidelines for the interpretation of sequence variants: A joint consensus recommendation of the American College of Medical Genetics and Genomics and the Association for Molecular Pathology. Genetics in Medicine 2015; 17: 405–424.

Ref. S2: Matisoo-Smith E. Ancient DNA and the human settlement of the Pacific: A review. *J Hum Evol*. 2015;79:93-104. doi:10.1016/j.jhevol.2014.10.017

Ref. S3: Fievet A, Mouret-Fourme E, Colas C, de Pauw A, Stoppa-Lyonnet D, Buecher B. Prevalence of Pathogenic Variants of FAN1 in More Than 5000 Patients Assessed for Genetic Predisposition to Colorectal, Breast, Ovarian, or Other Cancers. *Gastroenterology*. 2019;156(6):1919-1920. doi:10.1053/j.gastro.2019.01.003

STROBE Statement—checklist of items that should be included in reports of observational studies

	Item No.	Recommendation	Page No.	Relevant text from manuscript
**Title and abstract**	1	(*a*) Indicate the study's design with a commonly used term in the title or the abstract	3	"In this retrospective study…"
		(*b*) Provide in the abstract an informative and balanced summary of what was done and what was found	3	NA
**Introduction**				
Background/rationale	2	Explain the scientific background and rationale for the investigation being reported	4	Paragraphs 1 and 2
Objectives	3	State specific objectives, including any prespecified hypotheses	4	"Here, we report the high…"
**Methods**				
Study design	4	Present key elements of study design early in the paper	5	In this retrospective observational study, we included…"
Setting	5	Describe the setting, locations, and relevant dates, including periods of recruitment, exposure, follow-up, and data collection	5	Paragraph 1
Participants	6	(*a*) *Cohort study*—Give the eligibility criteria, and the sources and methods of selection of participants. Describe methods of follow-up<br>*Case-control study*—Give the eligibility criteria, and the sources and methods of case ascertainment and control selection. Give the rationale for the choice of cases and controls<br>*Cross-sectional study*—Give the eligibility criteria, and the sources and methods of selection of participants	5	Paragraph 1
		(*b*) *Cohort study*—For matched studies, give matching criteria and number of exposed and unexposed<br>*Case-control study*—For matched studies, give matching criteria and the number of controls per case		
Variables	7	Clearly define all outcomes, exposures, predictors, potential confounders, and effect modifiers. Give diagnostic criteria, if applicable	5	Paragraph 2
Data sources/ measurement	8*	For each variable of interest, give sources of data and details of methods of assessment (measurement). Describe comparability of assessment methods if there is more than one group	*5*	*Paragraph 2*
Bias	9	Describe any efforts to address potential sources of bias		NA

Study size	10	Explain how the study size was arrived at	NA

Continued on next page

Quantitative variables	11	Explain how quantitative variables were handled in the analyses. If applicable, describe which groupings were chosen and why	NA	
Statistical methods	12	(*a*) Describe all statistical methods, including those used to control for confounding	5	Paragraph 3
		(*b*) Describe any methods used to examine subgroups and interactions	NA	
		(*c*) Explain how missing data were addressed	NA	
		(*d*) *Cohort study*—If applicable, explain how loss to follow-up was addressed<br>*Case-control study*—If applicable, explain how matching of cases and controls was addressed<br>*Cross-sectional study*—If applicable, describe analytical methods taking account of sampling strategy	NA	
		(*e*) Describe any sensitivity analyses		
**Results**				
Participants	13*	(a) Report numbers of individuals at each stage of study—eg numbers potentially eligible, examined for eligibility, confirmed eligible, included in the study, completing follow-up, and analysed	7	"KIN was identified in twelve…"
		(b) Give reasons for non-participation at each stage	NA	
		(c) Consider use of a flow diagram	NA	
Descriptive data	14*	(a) Give characteristics of study participants (eg demographic, clinical, social) and information on exposures and potential confounders	Table 1	
		(b) Indicate number of participants with missing data for each variable of interest		
		(c) *Cohort study*—Summarise follow-up time (eg, average and total amount)	NA	
Outcome data	15*	*Cohort study*—Report numbers of outcome events or summary measures over time	7	"Kidney outcomes"
		Case-control study—Report numbers in each exposure category, or summary measures of exposure		
		Cross-sectional study—Report numbers of outcome events or summary measures		
Main results	16	(*a*) Give unadjusted estimates and, if applicable, confounder-adjusted estimates and their precision (eg, 95% confidence interval). Make clear which confounders were adjusted for and why they were included	NA	
		(*b*) Report category boundaries when continuous variables were categorized	NA	
		(*c*) If relevant, consider translating estimates of relative risk into absolute risk for a meaningful time period	NA	

Continued on next page

Other analyses	17	Report other analyses done—eg analyses of subgroups and interactions, and sensitivity analyses		
**Discussion**				
Key results	18	Summarise key results with reference to study objectives	9-10	
Limitations	19	Discuss limitations of the study, taking into account sources of potential bias or imprecision. Discuss both direction and magnitude of any potential bias	9	"Comparison between *FAN1* haplotypes of these Maori patients and those included in our series could not be performed…"
Interpretation	20	Give a cautious overall interpretation of results considering objectives, limitations, multiplicity of analyses, results from similar studies, and other relevant evidence	9-10	3 Because kidney biopsy was only performed at the time the patient was referred for, whether the progression of karyomegalic cells number within kidneys correlates with kidney outcomes could not be addressed. 3
Generalisability	21	Discuss the generalisability (external validity) of the study results		
**Other information**				
Funding	22	Give the source of funding and the role of the funders for the present study and, if applicable, for the original study on which the present article is based	1	

*Give information separately for cases and controls in case-control studies and, if applicable, for exposed and unexposed groups in cohort and cross-sectional studies.

Note: An Explanation and Elaboration article discusses each checklist item and gives methodological background and published examples of transparent reporting. The STROBE checklist is best used in conjunction with this article (freely available on the Web sites of PLoS Medicine at http://www.plosmedicine.org/, Annals of Internal Medicine at http://www.annals.org/, and Epidemiology at http://www.epidem.com/). Information on the STROBE Initiative is available at www.strobe-statement.org.
